# Novel Organically Modified Core-Shell Clay for Epoxy Composites—“*SOBM Filler 1*”

**DOI:** 10.1155/2014/282817

**Published:** 2014-10-29

**Authors:** Nnamdi Chibuike Iheaturu, Innocent Chimezie Madufor

**Affiliations:** ^1^Department of Polymer & Textile Engineering, Federal University of Technology, PMB 1526, Owerri, Imo, Nigeria; ^2^AppliedSignals International Research Group, Opposite Catholic Bishop's House, Maria Mater Ecclesia Cathedral, Nnarambia-Ahiara, Ahiazu-Mbaise, P.O. Box 326, Owerri, Imo, Nigeria

## Abstract

Preparation of a novel organically modified clay from spent oil base drilling mud (SOBM) that could serve as core-shell clay filler for polymers is herein reported. Due to the hydrophilic nature of clay, its compatibility with polymer matrix was made possible through modification of the surface of the core clay sample with 3-aminopropyltriethoxysilane (3-APTES) compound prior to its use. Fourier transform infrared (FT-IR) spectroscopy was used to characterize clay surface modification. Electron dispersive X-ray diffraction (EDX) and scanning electron microscopy (SEM) were used to expose filler chemical composition and morphology, while electrophoresis measurement was used to examine level of filler dispersion. Results show an agglomerated core clay powder after high temperature treatment, while EDX analysis shows that the organically modified clay is composed of chemical inhomogeneities, wherein elemental compositions in weight percent vary from one point to the other in a probe of two points. Micrographs of the 3-APTES coupled SOBM core-shell clay filler clearly show cloudy appearance, while FT-IR indicates 25% and 5% increases in fundamental vibrations band at 1014 cm^−1^ and 1435 cm^−1^, respectively. Furthermore, 3-APTES coupled core-shell clay was used to prepare epoxy composites and tested for mechanical properties.

## 1. Introduction

Clay is a general term including many combinations of one or more clay minerals with traces of metal oxides and organic matter. Geologic clay deposits are mostly composed of phyllosilicate minerals containing variable amounts of water trapped in the mineral structure [1]. In Nigeria, smectites, kaolinites, calcites, layered double hydroxides, and other mixed oxide clays are located within the formation in the earth's crust [2]. Furthermore, clay in its natural form is hydrophilic and organophobic, which makes dispersion and compatibility of mineral clay fillers in organic matrices difficult. Only a few hydrophilic polymers such as polyethylene oxide, poly vinyl acetate, and polyvinyl alcohol can be miscible with clay filler materials with less difficulty [3, 4]. However, in order to mitigate this setback in the use of clays in polymer matrices, functionalization of the clay platelets with organic compounds is one sure way. It has been reported that natural and synthetic clay modifications using organic compounds have been successful with aminosilanes [5, 6], organofunctional alkoxysilane [7], and alkylammonium compounds [8, 9]. On the other hand, natural smectite clays have been modified into porous clay structure, otherwise,* “core”* clay by various synthesis routes [10, 11] before grafting with organosilanes [12] and then inclusion in a host polymer matrix. In all the accounted cases, the ultimate reason for clay modification is to improve on particle dispersion and compatibility with host polymer by coating the clay with a soft organic compound, otherwise, an organic* “shell”* [10].  Table 1 is a list of organosilane compounds used in clay modification.

Furthermore, the effective dispersion and distribution of silane coupled core-shell clay fillers in polymer matrices, which usually enhances filler-polymer interaction, provokes significant improvement in material properties: mechanical, thermal, and optical properties. This is vital when preparing nanoparticulate reinforced functionally graded polymer composites. To be more specific, such particulate* core-shell clay* fillers also enhance diffusion of stress concentrations within the host matrix, thereby enhancing mechanical properties of their host matrix [13–16].

### 1.1. Core-Shell Clay Morphology

**Figure 1:**
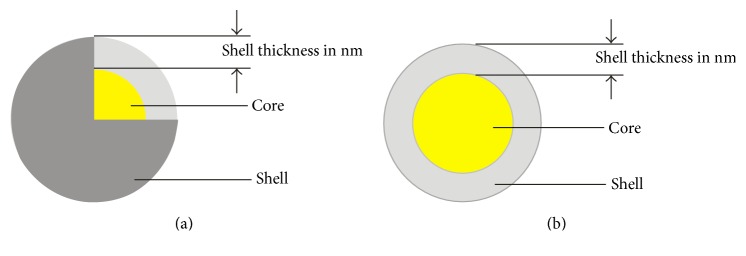
Schematic representation of core-shell morphology (a) with 1/8th quartet cut-off. (b) Transverse face of one half cut-off.

Core-shell nanoparticles may consist of inorganic or organic core coated with a thin film of inorganic or organic shell to form hybrid nanofillers: ORMOSILS or class I/II ORMOCERS [17, 18]. The inorganic core maybe natural or synthetic nanoclays: bentonite, silica, titania, zirconia or calcite, ferrite, alumina, hectorite, double layered hydroxides, or a mixture of clays [19–21]. The shell may be inorganic or organic/polymers with functionalities [22]. They may exhibit combination of qualities which neither are inherent in the core nor in the shell.  Figure 1 shows schematic representations of core-shell nanofiller morphology.

However, procedures for obtaining core-shell nanoparticles have been elucidated by [23, 24]. Core-shell nanoparticles have found applications in polymer nanocomposites, coatings and adhesives, optics, electronics, biomedical assays, catalysis, industrial sieves, and environmental applications [24].

In this study, our aim is to prepare and report on the characterization of novel organically modified* “core-shell clay”* filled epoxy composites from spent drilling mud.

## 2. Experimental

**Figure 2:**
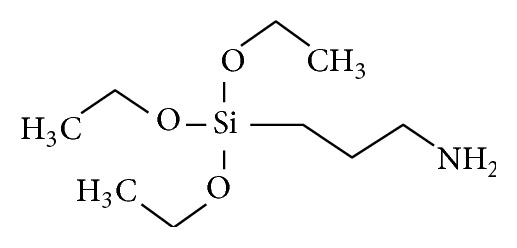
Structural formula of 3-aminotriethoxysilane (3-APTES). Linear formula: H_2_N(CH_2_)_3_Si(OC_2_H_5_)_3_, molecular weight: 221.37 g, density: 0.946 g/mL at 25°C, and boiling point: 217°C/760 mmHg.

Core clays used in this study have been synthesized from spent oil-base drilling mud and earlier reported [25]. The epoxy resin used was a two- [2] component laminating PRO-SET epoxy system from PRO-SET Inc., Bay City, MI, USA. The components are 135/229 epoxy resin and phenol-free hardener, which are formulated for laminating synthetic composite structures. Such composites are produced for high performance, light weight structures using woven, multiaxial glass fabrics, aramid, carbon, and hybrids. The 135/229 mixture provides a working time of approximately 120 minutes at 22.2°C bonding. According to ASTM D-2427-71, 135/229 has pot-life of 59 minutes for 100 g mixture. All chemicals used for this work were of analytical grade. Acetone (CH_3_COCH_3_) and tetraoxosulphate VI acid (H_2_SO_4_) were obtained from MERCK KGaA, Darmstadt, Germany. Anhydrous ethanol, 99%, was gotten from Kemetyl A/S, Køge, Denmark. Hexadecyltrimethylammonium bromide (HDTMA-Br) (C_19_H_42_N·Br) dispersant was from SIGMA-ALDRICH, while 3-Aminopropyltriethoxosilane (3-APTES), with structural formula shown in Figure 2, was supplied by Alfa Aesar GmBH & Co. KG, Karlsruhe, Germany.

### 2.1. Method for Silane Coupling

**Figure 3:**
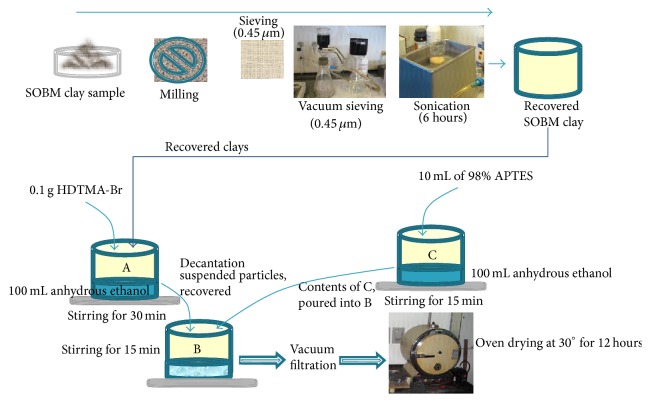
Schematic of the process of silane coupling.

Nanoporous clay powder sample from solvothermal synthesis and calcination of spent oil-base mud (SOBM) were ground in a ceramic mortar and sieved into various sizes. From cumulative frequency curves, the least size of 63 *μ*m which is the effective particle size and maximum particle size of the smallest 10.95%. SOBM powder material was sonicated in an ultrasonic bath for 6 hrs with 58 gmol^−1^ acetone to clean the pores in the samples, then filtered, and dried. Then, 0.1 g of hexadecyltrimethylammonium bromide (HDTMA-Br) was poured into Beaker A containing 100 mL of anhydrous ethanol with continuous stirring for 30 minutes. HDTMA-Br is a cationic surfactant which would repel H^+^ ions on the clays as a result of acid treatment, thereby suspending the particles. After a while, heavier particles in the mixture settled at the bottom of the beaker, while suspended particles were recovered by decantation. 10 mL of 98% 3-Aminopropyltriethoxysilane (APTES) was put into another Beaker, C, containing 100 mL of anhydrous ethanol. After allowing the mixture of HDTMA-Br/clay in ethanol to stir for another 30 minutes, contents of Beaker A and Beaker C, were poured into a third Beaker, B, and altogether stirred for 15 minutes. This process was used to disperse the clay samples while grafting silane end groups from 3-APTES to the core clay bodies. Afterwards, the mixture was vacuum filtered with Durapore polypropylene membrane filters HVLP 0.45 *μ*m sieve size to recover the clay powder. The clay powder was dried in an oven at 30°C for 12 hours and then stored in a desiccator.  Figure 3 presents a schematic of the process of silane coupling from grinding to sieving, ultrasonication, silane coupling, and oven drying.

### 2.2. Scanning Electron Microscopy (SEM) and Chemical Analysis by EDX

The Zeiss Scanning Electron Microscope, model: EVO 60, with 10 KV accelerating voltage, was used for SEM/EDX analysis to examine the clay microstructure. Energy dispersive X-ray scattering (EDX) exposed sample chemical composition. 63 *μ*m size SOBM core clay particles were subjected to EDX analysis in order to expose core clay chemical composition as well as level of chemical homogeneity in a 2-point analysis.

### 2.3. X-Ray Diffraction (XRD) Analysis

In order to determine mineralogy, basal *d*-spacing, and crystallography of powder samples, XRD pattern of the sample was obtained with a Philips PW1050 X-ray Diffractometer using Cu-K*α* radiation of wavelength 1.54 Å, voltage 40 kV, and 50 mA. Scanning was done from 1° to 80° (2*θ*) in steps of 0.05 and 1 sec count time per step.

#### 2.3.1. Zeta Potential Measurement of Clay Particles

Zeta potential measurement on clay powder was carried out at 25°C with DELSA 440SX electrophoresis equipment. Various concentrations of the clay samples were prepared in potassium chloride (KCl) electrolyte medium kept constant at 10^−3^ M. The sample pH was increased using a base, sodium hydroxide (NaOH), and reduced using an acid, hydrochloric acid (HCl). The samples were allowed to equilibrate for 2 hours before zeta potential readings in millivolts (mV) were taken.

### 2.4. Fourier Transform Infrared Spectroscopy (FTIR) on 3-APTES Coupled SOBM Core Clay

Fourier transform infrared spectrophotometer from Bruker Optics was used to determine the silane coupling on the core clay material. 100 scans of powder samples were performed in attenuated total reflectance (ATR) transmittance mode, beam splitter; KBr, Globar lamp detector of DTGS/KBr.

#### 2.4.1. Method for Epoxy Composite Moulding

For epoxy composite preparation, pure samples were produced without inclusion of 3-APTES coupled SOBM core-shell filler. Then, epoxy composite formulation with various weight fractions, 0.8 wt%, 1.6 wt%, 4.0 wt%, and 8.0 wt% of 3-APTES coupled SOBM core-shell filler corresponding to 1 g, 2 g, 5 g, and 10 g, was poured into 100 g of PRO-SET epoxy matrix and mechanically stirred for 30 mins. Then, 26 g of phenol free hardener was added to the epoxy clay mixture and stirred vigorously with a high speed mechanical mixer for 5 mins. The formulation mixture of 100 g epoxy and 26 g hardener with different 0, 0.8, 1.6, 4.0, and 8.0 weight percent of 3-APTES coupled clay was poured into dog bone shaped silicone mould in order to obtain epoxy-core-shell-clay composites for testing. The composite samples were allowed to cure for 48 hrs before releasing them from the mould.

#### 2.4.2. Tensile Testing of 3-APTES Coupled Core-Clay Filled Epoxy Composites—ASTM D 638

Tensile test was carried out with ZWICK/ROELL Z100 equipment according to ASTM D 638. Testing was carried out with a cross-head speed of 500 *μ*m/min; grip-to-grip separation was 60 mm while E-modulus speed was 2 mm/min. The E-modulus was electronically measured by the extensometer which was directly connected to the dumb bell sample. All measurements were zeroed before testing.

## 3. Results and Discussions

### 3.1. SEM/EDX Analysis of Core Clay Filler

Generally, a clear case of chemical inhomogeneity in the SOBM clay is evident in the two-point analysis and standard deviation calculations shown in Table 2. This is a general problem associated with clay samples of natural origin where chemical composition is not easily controlled, unlike the synthetic clays whose chemical compositions can be controlled ab initio from initial precursors. Disparity or deviation from mean value of element composition in weight percent (Wt %) is shown as standard deviation also measured in weight percent. The deviation however depends on specific point on the powder scanned at a given time. Calcium (Ca) differed greatly from one point to the other by showing a standard deviation of 15.08 weight percent, followed by iron (Fe) with a standard deviation of 5.48 weight percent.

### 3.2. Micrographs of Sonicated and Synthesized SOBM Core Clay

**Figure 4:**
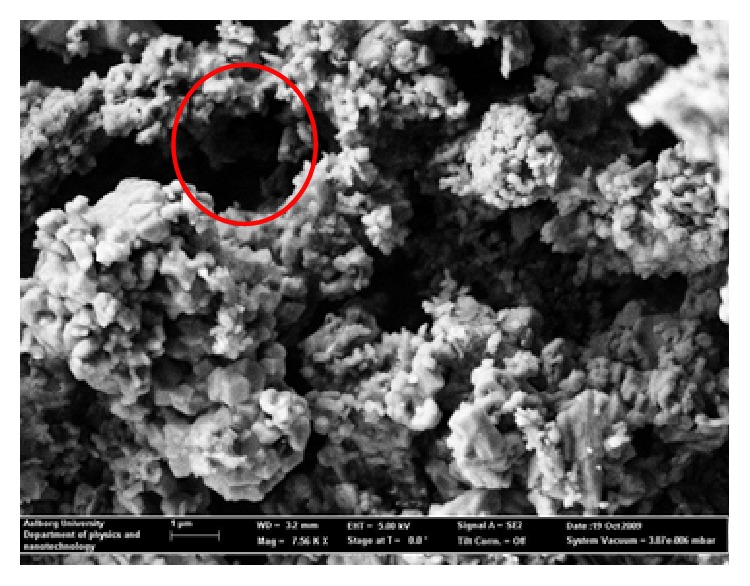
Sonicated and synthesized SOBM clay.

**Figure 5:**
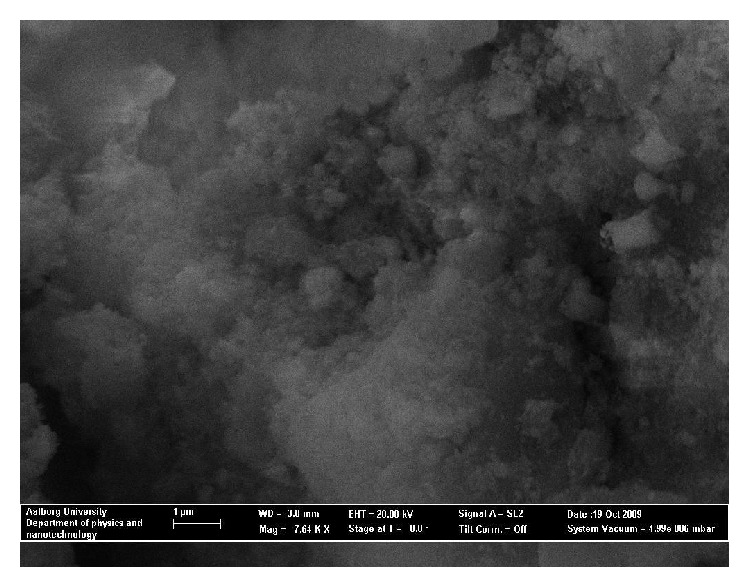
3-APTES coupled SOBM clay.

Scanning electron micrographs of the samples are shown in Figures 4 and 5.

With the same resolution, micrograph of sonicated and synthesized SOBM clay presents a highly porous material. With ultrasonication, the pores may have been cleaned of dusts and impurities which could act as blockages within the pore zones. Also, it can be observed that clay agglomerates are strongly held together and particles are glued to each other as a result of high temperature treatment. Each agglomerate zone can be said to be composed of a number of nanoparticles of size less than 1 *μ*m from the resolution measurement. The only way such agglomerates can be broken up may be by communition involving any of ball milling, jaw milling, crushing, or grinding. Points of viscous flow of melt due to high temperature treatment at 900°C are also visible. Points of contact between clay particles may have resulted in thin melt regions of complex oxides or spinel regions.

#### 3.2.1. Micrograph of 3-APTES Coupled SOBM Clay

Micrograph of 3-APTES coupled SOBM clays is presented in Figure 5. The figure presents a cloudy appearance of clay powder sample. The cloudy image can be attributed to 3-APTES coupling which appear as white silanol compound precipitated on the clay bodies after hydrolysis and condensation within hydrocalcite and hydrous aluminosilicate regions.

Figure 5 shows a powder material that is coated with a cloudy appearance. The cloudy appearance may be attributed to silane polycondensed silanol attachments on the clay bodies. It is possible that the material would be nonconductive which may be a result of silanol linkages on the synthesized SOBM clay. Very high magnification, 7.64 Kx, was only able to expose a few pores within the clay bulk.

#### 3.2.2. Results of XRD of SOBM Core Clay Sample

**Figure 6:**
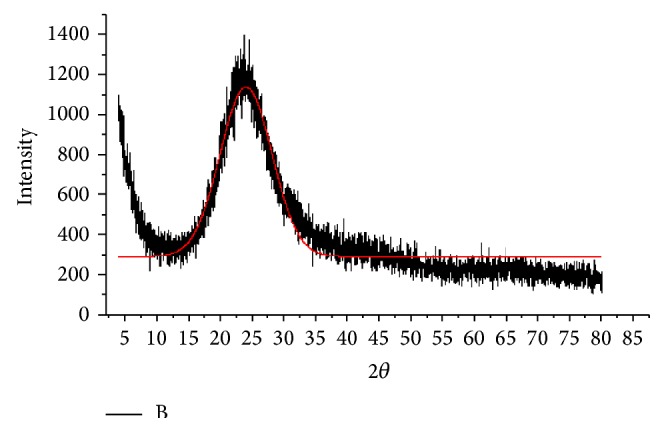
X-ray diffraction result of synthesized SOBM powder showing plot of intensity in a.u. against diffraction angle of 2*θ*.

X-ray diffraction result presented in Figure 6 shows only one peak with a broad peak width.

However, the increase at the lower angle, between 0° and 10° 2*θ*, represents the incident beam primarily from the X-ray tube directly into the detector. The main peak at 25° diffraction angle, 2*θ*, is broad which suggests that the material under this X-ray investigation should be amorphous wherein there seems to be a complete loss of clay crystallographic pattern. Only amorphous materials like glasses without regular crystallographic patterns can present broadband X-ray diffraction patterns. Also thin films in which case only one orientation gives peak related to the dimension of the film layer structure can also give this type of pattern. From Figure 6,* D*_001_ spacing shows neither intercalation nor exfoliation, rather it shows that the clay platelets interparticle space is 0.37 nm in maximum length. This means that clay particles are either agglomerated or are being held together by aluminosilicate nanobridges. This presents another clear evidence of nanoporosity of the clay particles.

#### 3.2.3. Results of Electrophoresis Experiment on Core Clay Sample

**Figure 7:**
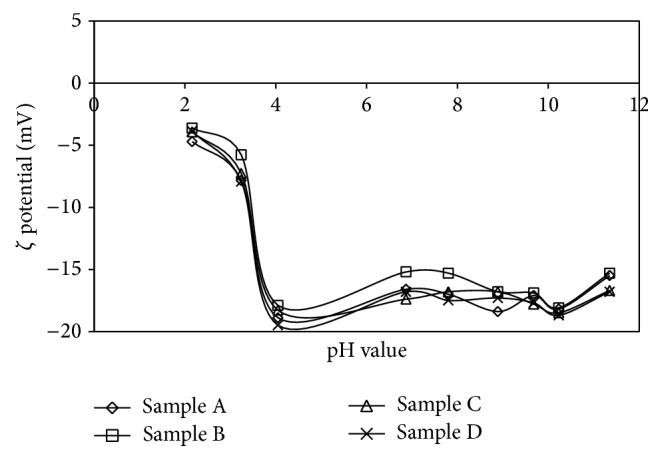
Electrophoretic measurements of synthesized SOBM and thermally desorbed clay samples.

Results of electrophoresis measurements for SOBM clay samples A, B, C, and D are presented in Figure 7.

The results as compared for samples A, B, C, and D show the same graph patterns. No clear point of zero charge (PZC) was noticed as the particles did not show any reasonable sign of dispersion. Zeta potential of 0 to ±5 mV and ±10 to ±30 mV confirms flocculated particles that are heavy, porous at the same time poorly charged. This confirms the SOBM samples to be agglomerated, porous powder which may be as a result of poorly separated particles during solvothermal treatment. However, the specific gravity of the powder, 2.77 gcm^−3^ as earlier reported by Enyiegbulam et al. [25], presents heavy particles that would rather sink rapidly than maintain a stable, dispersed colloidal system. The isoelectric points (IEP) went from −3 to −18 for the core (porous) clay material. There is enough reason to recommend further breakup of particles thereby reducing particle size and increasing surface area of the particles. This can be achieved by communition, milling, or jaw crushing.

### 3.3. FT-IR Spectra of 3-APTES Coupled SOBM Core-Shell Clay Filler

**Figure 8:**
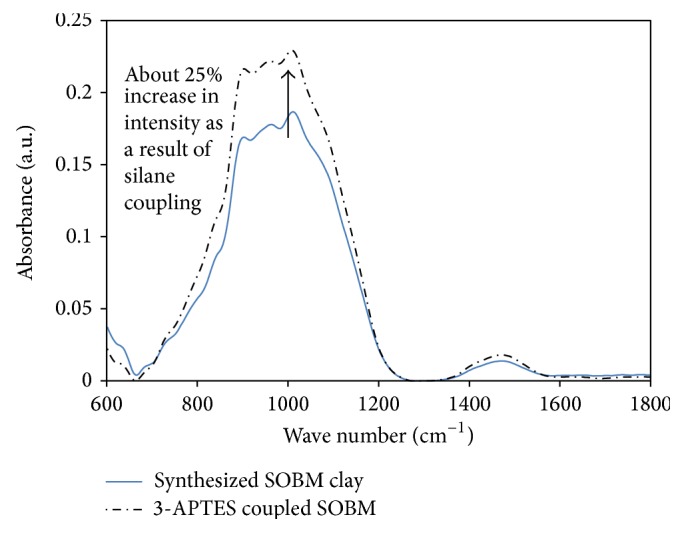
FT-IR spectra of synthesized, sonicated, and 3-APTES coupled spent oil base drilling mud (SOBM) core clay in absorbance mode.

FT-IR spectra of spent oil-base drilling mud (SOBM) samples calcined at 900°C present broad band at 530 cm^−1^, 550 cm^−1^, 640 cm^−1^, 872 cm^−1^, 1030 cm^−1^, 1208 cm^−1^, and 1366 cm^−1^. The synthesized SOBM core clay must have been an amorphous silicate/hydrocalcite mixed oxide clay, with broadband IR spectrum due to a complete disorder in silicate chains arrangement, whereby O–Si–O chains have been frozen during cooling. Dislocation of Al by Fe within the lattice of silicates layers must have contributed immensely to egg-yolk-yellow colour change of powder sample, while Ca^2+^ ions remained within the interlayer spacing. These cations must have been actively involved in adsorbing silane chains of 3-aminopropyltriethoxysilane coupling agent to the clay bodies. FT-IR spectra of synthesized SOBM clay and the 3-APTES coupled SOBM core-shell clay samples are both plotted on one graph in Figure 8.

The spectra show clear increase in intensity of fundamental vibrations, *V*_*b*_, of O–Si–O bond at 1014 cm^−1^, and –CH_2_– asymmetric stretching vibrations, *V*_asy_, at 1435 cm^−1^, by at least 25% and 10%, respectively. Reason adduced for this is that there may have been an increase in hydration level as a result of structural OH group, which leads to increase in silane coupling on the nanoporous SOBM core clay platelets. Interestingly, the intense spectra of sonicated calcined SOBM sample had increased to higher degrees up to approximately 20% after silane coupling. There were no new peaks generated or overtones as a result of sonication or silane coupling.

### 3.4. Results of Tensile Testing of Epoxy Composite Samples (ASTM D-638)

**Figure 9:**
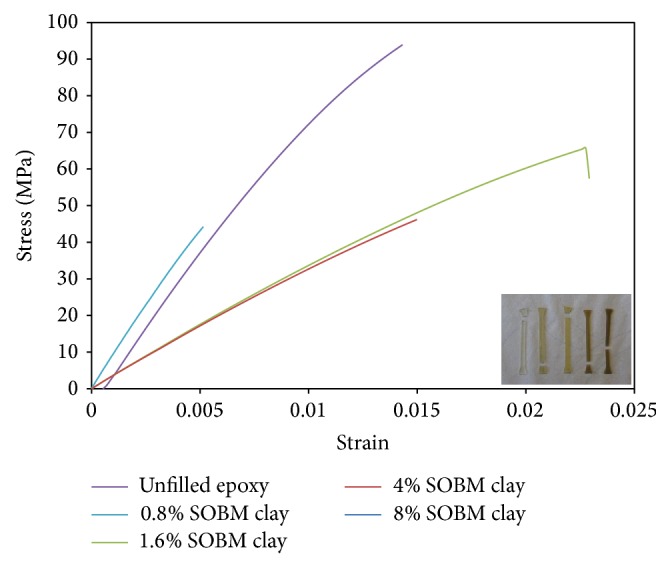
Comparative stress-strain behaviour of 3-APTES coupled SOBM core-shell clay filled epoxy composites. Insert shows the five tested composite samples, showing points of fracture.

A comparative investigation of tensile strength of epoxy composite samples as plotted in Figure 9 indicates relatively stiff composite materials, wherein considerable resistance to deformation can be observed in all 3-APTES coupled SOBM core-clay filled epoxy composites. Most of the composites showed very low stress and low strain rate except the unfilled epoxy.  Table 3 is a tabulation of strain, maximum displacement, ultimate strength, and E-modulus of 3-APTES coupled SOBM core-clay filled epoxy composites.

Table 3 is a tabulation of strain, maximum displacement, ultimate strength, and E-modulus of 3-APTES coupled SOBM core-shell clay filled epoxy composites obtained from Figure 9.

0.8 wt% or 1 g of 3-APTES coupled SOBM core-shell clay filler had shown the lowest stiffness with E-modulus of 896.1 MPa. But as filler content increased from 1.6 wt% or 2 g to 4 wt% or 5 g in the epoxy composite, E-modulus increased from 3.135 GPa to 3.253 GPa. Increased 3-APTES coupled SOBM core-shell clay filler content from 4 wt% to 8 wt% or 10 g gave a composite with E-modulus 3.093 GPa. Displacement of the composite material appears to be very low for 0.8 wt% 3-APTES coupled SOBM core-shell clay filled epoxy composite, which is indicative of a very weak and brittle epoxy composite material. Even though 0.8 wt% 3-APTES coupled SOBM core-shell clay filled epoxy may be described as being weak and brittle, its behaviour could be likened to the unfilled epoxy having low ultimate strength. On the other hand, 1.6 wt% 3-APTES coupled SOBM core-shell clay with maximum displacement of 1.254 mm and ultimate strength of 65.732 MPa, presents a relatively tough composite material. Comparatively, with higher filler content above 1.6 wt% or 2 g, epoxy composite became more plastic. This may be attributed to the porous nature of the 3-APTES coupled SOBM core-shell clay filler material.

#### 3.4.1. Effect of Silane Coupled (Core-Shell) Fillers on Mechanical Properties of Epoxy Composites

**Figure 10:**
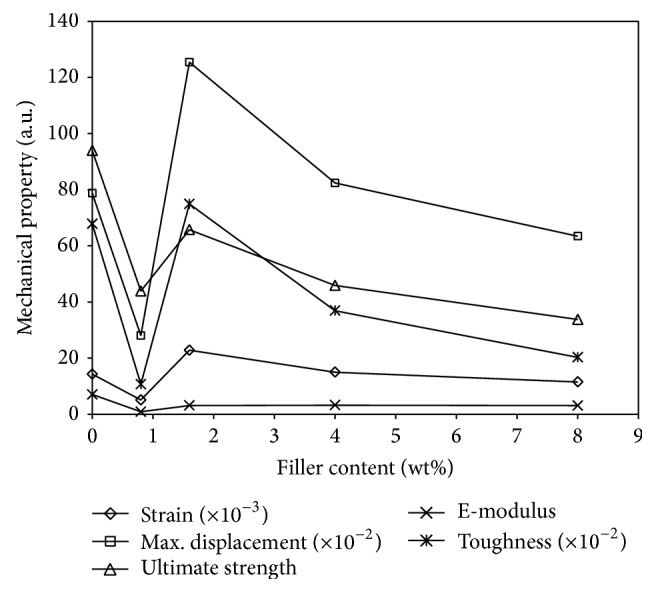
Comparative plot showing the effect of 3-APTES coupled SOBM core clay filler content on the mechanical properties of epoxy composites.

The effects of filler content of 3-APTES coupled SOBM core-shell clay fillers on the mechanical properties of epoxy composites are revealed in Figure 10. The plotted values were extracted from Figure 9 and presented in Table 3. The mechanical properties as ultimate stress, toughness, E-modulus, and maximum displacement are plotted on the *y*-axis against filler content in weight percent (wt%) of core-shell clay filler in epoxy matrix on the *x*-axis.

Figure 10 shows that all mechanical properties of the epoxy composite decreased with 0.8 wt% 3-APTES coupled SOBM core-shell clay filler, but increased tremendously when filler content was doubled to 1.6 wt%. The continuous increase of the 3-APTES coupled core-shell clay filler in the epoxy matrix did not yield any more positive results in improved toughness, modulus, ultimate stress, maximum displacement, and strain.

## 4. Conclusion

Organically modified calcite fillers synthesized from drilling mud solid content (clay) in an oil base were organically modified with 3-aminopropyltriethoxysilane (3-APTES) which formed a soft shell on the nanoporous core powder. Interestingly, it has been shown by FT-IR spectroscopy that there were about 25% and 5% increases, respectively, in intensity of fundamental vibrations, *V*_*b*_, of O–Si–O bond at 1014 cm^−1^, and –CH_2_– asymmetric stretching vibrations, *V*_asy_, at 1435 cm^−1^. This intensity shows actively bonded O–Si–O linkages forming a soft shell coating as a result of polycondensation reactions with structural OH groups on the nanoporous clay bodies. It has also been possible to show via 2-point EDX analysis that the clay powder material does not have a homogeneous chemical composition, a characteristic of clays from natural origin while, as a result of solvothermal treatment of the natural clays, there may have been a complete or near collapse of the layer structure of the phyllosilicate clays present in the spent drilling mud sample leading to the formation of nanobridges. Interlayer distance was measured as 0.37 nm. Only amorphous materials like glasses without regular crystallographic patterns can present broadband diffraction patterns. The novel filler material, *“SOBM Filler 1,”* may be regarded as organically modified calcite (ORMOCAL) filler, which may be used as alternative filler material for polymers: thermoplastics and thermosets. However, all mechanical properties of the epoxy composite decreased with 0.8 wt% 3-APTES coupled SOBM core-shell clay filler but increased tremendously when filler content was doubled to 1.6 wt%. The continuous increase of the 3-APTES coupled core-shell clay filler in the epoxy matrix did not yield any more positive results in improved toughness, modulus, ultimate stress, maximum displacement, and strain.

## Figures and Tables

**Table 1:** List of some organosilane compounds for clay surface modification.

Organofunctional silane	Silane chemical formula
n-Octadecyltriethoxysilane	(OC_2_H_5_)_3_Si(CH_2_)_17_CH_3_
n-Octadecylmethyldiethoxysilane	(OC_2_H_5_)_2_CH_3_Si(CH_2_)_17_CH_3_
n-Octadecyldimethylmethoxysilane	(OC_2_H_5_)_1_(CH_3_)_2_Si(CH_2_)_17_CH_3_
3-Aminopropyltriethoxysilane	(OC_2_H_5_)_3_Si(CH_2_)_3_NH_2_
3-Aminopropylmethyldiethoxysilane	(OC_2_H_5_)_2_CH_3_Si(CH_2_)_3_NH_2_
3-Aminopropyldimethylethoxysilane	(OC_2_H_5_)_1_(CH_3_)_2_Si (CH_2_)_3_NH_2_

**Table 2:** Two-point analysis of synthesized untreated SOBM clay filler.

Point 1					Point 2					Standard deviation (Wt%)
Element	Atomic Wt (g)	Wt%	% Atom	Number of mols. of element	Element	Atomic Wt (g)	Wt%	% Atom	Number of mols. of element	
O	16	37,94	62,73	2,4	O	16	9,20	21,95	0,6	20,32
Na	23	0,39	0,45	0,0	Na	23	—	—	0,0	—
Al	27	4,88	4,78	0,2	Al	27	3,30	4,66	0,1	1,12
Si^*^	28	15,9	14,97	0,6^*^	Si^*^	28	16,78	22,81	0,6^*^	0,62^*^
S	32	1,81	1,49	0,1	S	32	0,55	0,66	0,0	0,89
Cl	35	0,27	0,2	0,0	Cl	35	—	—	0,0	—
K	39	1,38	0,94	0,0	K	39	3,29	3,21	0,1	1,35
Ca^**^	40	13,47	8,89	0,3^**^	Ca	40	34,79	33,14	0,9^**^	15,08^**^
Ti	—	—	—	—	Ti	48	0,30	0,24	0,0	—
Fe	56	3,29	1,56	0,1	Fe	56	11,04	7,55	0,2	5,48
Ba	137	20,68	3,98	0,2	Ba	137	20,74	5,76	0,2	0,04

**Table 3:** Mechanical properties of 3-APTES coupled-SOBM core clay epoxy composites.

SOBM epoxy sample ID (weight %)	Strain	Max. displacement (mm)	Ultimate strength (MPa)	Estimated stiffness (E-modulus in GPa)
SOBM 0	0.0143	0.787	93.91	7.065
SOBM 0.8	0.0051	0.2805	43.875	0.8961
SOBM 1.6	0.0228	1.254	65.732	3.135
SOBM 4	0.01498	0.8239	45.889	3.253
SOBM 8	0.01153	0.6342	33.773	3.0929
